# Longitudinal Analysis of Image Time Series with Diffeomorphic Deformations: A Computational Framework Based on Stationary Velocity Fields

**DOI:** 10.3389/fnins.2016.00236

**Published:** 2016-06-03

**Authors:** Mehdi Hadj-Hamou, Marco Lorenzi, Nicholas Ayache, Xavier Pennec

**Affiliations:** Asclepios Research Project, INRIA Sophia AntipolisSophia Antipolis, France

**Keywords:** deformation-based morphometry, non-linear registration, longitudinal study, diffeomorphism parametrized by stationary velocity fields, statistical analysis, reproducible research

## Abstract

We propose and detail a deformation-based morphometry computational framework, called Longitudinal Log-Demons Framework (LLDF), to estimate the longitudinal brain deformations from image data series, transport them in a common space and perform statistical group-wise analyses. It is based on freely available software and tools, and consists of three main steps: (i) Pre-processing, (ii) Position correction, and (iii) Non-linear deformation analysis. It is based on the LCC log-Demons non-linear symmetric diffeomorphic registration algorithm with an additional modulation of the similarity term using a confidence mask to increase the robustness with respect to brain boundary intensity artifacts. The pipeline is exemplified on the longitudinal Open Access Series of Imaging Studies (OASIS) database and all the parameters values are given so that the study can be reproduced. We investigate the group-wise differences between the patients with Alzheimer's disease and the healthy control group, and show that the proposed pipeline increases the sensitivity with no decrease in the specificity of the statistical study done on the longitudinal deformations.

## 1. Introduction

An important topic in neuroimaging is to analyse the progression of morphological changes in the brain observed in time series of images, in order to model and quantify normal or pathological biological evolutions (Scahill et al., 2002). Deformation-Based Morphometry (DBM) (Ashburner et al., 1998) characterizes the morphological changes of the brain in terms of spatial transformations (here called deformations), estimated by means of non-linear registration. A sub-field of DBM, called Tensor-Based Morphometry (TBM) focuses on the first derivatives of the deformation. Depending on the cross-sectional or longitudinal nature of the dataset used, we can define on one hand cross-sectional DBM and on the other hand longitudinal DBM (Chung et al., 2001) that we will focus on in this article. Longitudinal DBM main steps can be summarized as (i) quantifying the evolution of the morphology of each subject by estimating the individual's longitudinal deformation from the time series of images, and (ii) characterizing how this evolution varies among a sample using a suitable normalization for the individual biological variability. A variety of DBM approaches can be found in the literature (e.g., Davatzikos et al., 2001; Cardenas et al., 2007; Lorenzi et al., 2011; Südmeyer et al., 2012), each of them associated to specific non-linear registration methods, and processing pipelines. The comparison between the different DBM methods is not straightforward: the efficiency of each DBM pipeline is generally demonstrated on different data sets (or different subsets of the same data set) and the tools the processing pipeline is composed of are generally not all available. In the existing DBM pipelines—e.g., SPM (Friston, 2007), FreeSurfer (Reuter et al., 2012), PipeDream^1^, Anima^2^—the multivariate information coming from the three-dimensional deformation is generally not used for the statistical analysis. To do so, one would need to express the three-dimensional deformation of every subject in a common space to compare them. There exists few algorithms that compute this 3D transport (e.g., Lorenzi and Pennec, 2013) and in the absence of this tool, the DBM analysis often becomes a TBM analysis only. Studies are thus generally performed on the Jacobian determinant of the deformation or on the segmented regions of interest—since it is easier to compute these scalar maps in a common space. Moreover, in the developing context of reproducible research that has gained interest over the last years (Nature, 2013; McCormick et al., 2014), a good practice should be for researchers to publish the full details of their methodology: source code, data and parameters.

This is the objective of this article: to gather all the details in the same paper and propose a pipeline for the community, following the examples of Avants et al. (2011) and Ashburner and Ridgway (2013). Our computational framework is a complement to the existing processing pipelines. It enables researchers to replicate and verify their findings with a third party reproducible pipeline, thus enhancing the convincing power of their results. Our pipeline is based on Lorenzi et al. (2011), who proposed a hierarchical framework for the group-wise analysis of time series of images using diffeomorphic deformations parameterized by Stationary Velocity Fields (SVF). We bring a complement to the already existing literature by explicitly detailing all the processing steps required for the longitudinal analysis of neuroimages by relying on freely available tools. In addition to this contribution, we integrate a modification to the non-linear registration algorithm by adding a masking to the similarity term as proposed by Brett et al. (2001) while keeping the symmetry of the formulation. This change increases the robustness of the results with respect to intensity artifacts located in the brain boundaries. The proposed processing pipeline is based on freely available software and tools [the complete list can be found in Appendix (Supplementary Materials)].

The paper is structured as follows: in Section 2, we develop a comprehensive processing pipeline called Longitudinal Log-Demons Framework (LLDF); we present each elementary modules it is based on, and after introducing the mathematical formalism related to DBM, we modify the LCC log-Demons to incorporate a confidence mask. Experimental results show that this contribution leads to increased sensitivity of the statistical study on the longitudinal deformations. In Section 3, we show an illustration of the pipeline on the statistical analysis of longitudinal brain changes in Alzheimer's disease. Because it is freely and easily available for benchmarking, we use the data from the longitudinal Open Access Series of Imaging Studies (OASIS) database (Marcus et al., 2010). We finally conclude and present the perspectives of this work in Section 4.

## 2. Processing pipeline for the analysis of longitudinal images

**Figure 1:**
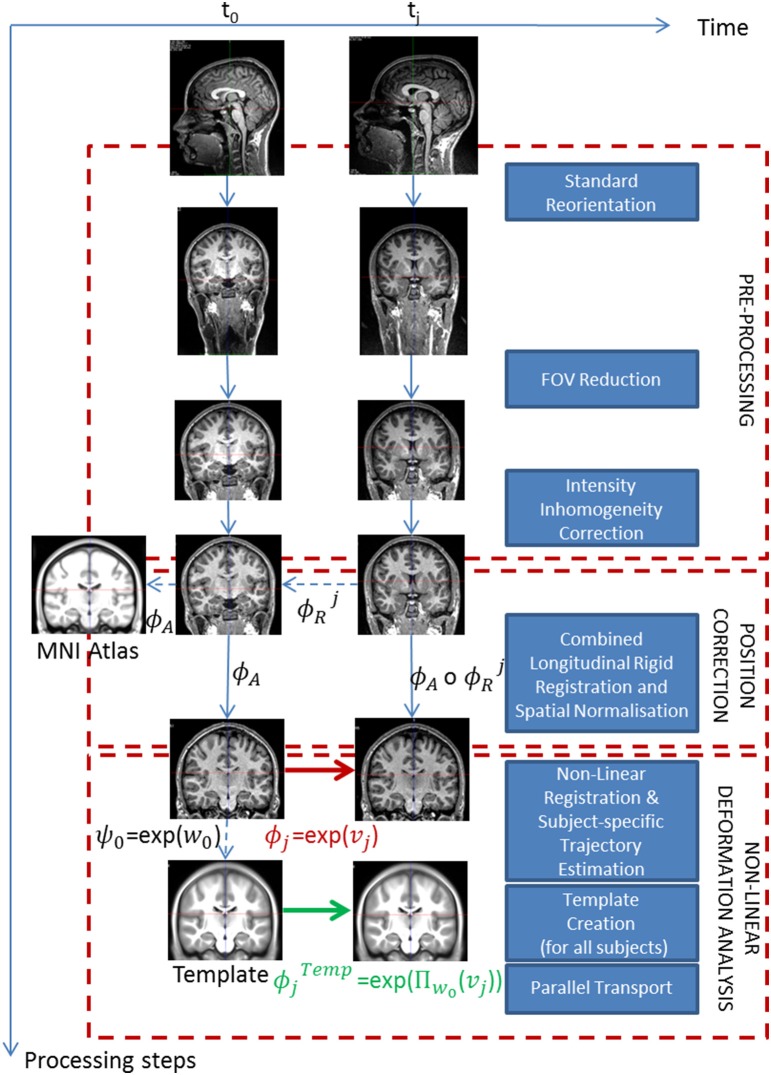
**Proposed processing pipeline for longitudinal analysis**. The pipeline is composed of three major steps. Starting with raw images, we first pre-process them, then correct the spatial position differences to end up with the longitudinal deformations for each subject in the template space. Dotted lines correspond to evaluated transformations whereas plain lines correspond to applied transformations.

We consider longitudinal observations of MRI scans for a given subject *S*_*i*_, at *N*_*i*_ time points *t*_0_, *t*_1_,…, *t*_*N*_*i*_−1_ (all the subjects do not necessarily have the same number *N*_*i*_ of time points). The corresponding images are denoted as $I_{0}^{i}$, $I_{1}^{i}$,…, $I_{N_{i}-1}^{i}$ respectively. The aim of the processing pipeline is to estimate each subject's longitudinal deformation from the image time series, and then transport the deformations in a common space to perform statistical group-wise analyses. The construction of the pipeline is based on elementary modules described in the following paragraphs and it can therefore be divided into three main parts (cf. Figure 1): (1) Pre-processing, (2) Position correction, and (3) Non-linear deformation analysis. The pipeline proposed in this work relies on a number of neuroimaging tools previously proposed and validated by different groups. Our choice was motivated by our personal experience and by the optimal performances obtained in the presented application. We however acknowledge that other tools could have been employed. For this reason, the modular nature of the pipeline allows the replacement of the proposed tools with specific ones, such as in the case of longitudinal analysis in postnatal brain development (cf. Section 2.1.3).

### 2.1. Pre-processing

In this initial part of the pipeline all the individuals' images are processed independently of the time points. The pre-processing consists of the following chain of elementary steps: (1) Standard reorientation, (2) Field of view reduction and, (3) Intensity non-uniformity correction. Different criteria have been taken into account for choosing the tools and software used to perform these elementary steps. Firstly, we only selected freely available tools part of well-established software—so that the pipeline can be reproduced by anyone—relying on already validated tools. Secondly, to make the pipeline user-friendly, we chose tools that necessitate minimal fine tuning in terms of parameters.

#### 2.1.1. Standard reorientation

**Figure 2:**
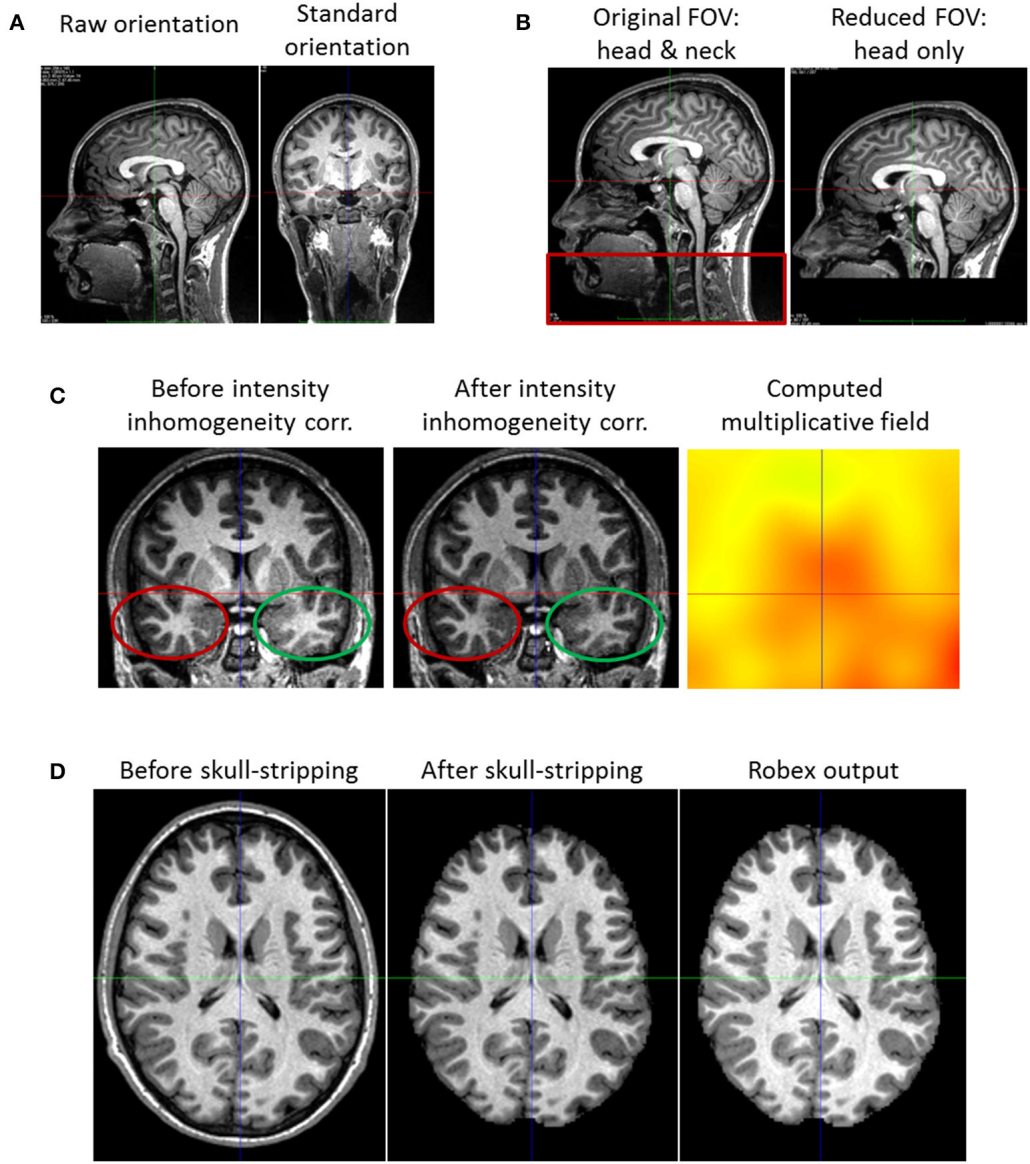
**Pre-processing steps**. **(A)** Reorientation of a subject coronal view. Left: what is displayed initially as the coronal view is the sagittal one. Right: after reorientation it is truly the coronal view that is displayed. **(B)** Field of View Reduction. Left: the original Field of View (FOV) including the head and neck (red rectangle). Right: after reduction, the cropped FOV does not contain the neck, but only the head. **(C)** Intensity Inhomogeneity Correction. Left: the image has an intensity non-uniformity. The same tissue class has a lower intensity in the bottom left (red ellipse), and a higher intensity in the bottom right part of the image (green ellipse). Middle: after correction, the intensity appearance of the image is more homogeneous (cf. red and green ellipses). Right: estimated multiplicative field. **(D)** Skull-stripping. Left: the head with its skull. Middle: the brain after the whole process of skull-stripping and image masking. We see that the resulting image has the same intensity as the original one; this is not the case of the image output by Robex (right image).

Images from the MRI scanner are not necessarily oriented following the standard orientation defined by the MNI152 (Fonov et al., 2009) template (Figure 2). This misorientation would prevent us from properly processing the images.

We thus use FSL - fslreorient2std (Jenkinson et al., 2012), to reorient each image to match the standard orientation. Starting with *I*, the image acquired by the scanner, this tool applies rotations of 0, 90, 180, or 270 degrees around the image axes to get *I*^*std*^, the reoriented output image. Notice that this reorientation only changes the header and does not perform any interpolation.

#### 2.1.2. Reduction of the field of view

Brain scans can sometimes include the neck or the shoulders (cf. Figure 2), and analysing the whole image would increase the image processing time and lead to increased errors due to intensity artifacts. Therefore, it is preferable to reduce the Field of View (FOV) of the image to include the head only.

For this purpose, we use FSL–robustfov (Jenkinson et al., 2012): given an image *I*, comprising the head and the neck, it automatically crops the neck and other regions outside the head by re-sizing the height of the image, starting at the top of the skull, to a default size of 170 mm so that we finally obtain *I*^*head*^, the image containing the head only.

In some rare cases (in another study not reported here, one case out of 120), this automatic tool might provide a wrong result, leaving an important part of the neck in the image or cropping the head. In that case, one can still manually set the correct height of the head.

#### 2.1.3. Intensity inhomogeneity correction

One of the most common artifact in MRI scans is the shading one: an intensity non-uniformity for voxels of the same tissue class (cf. Figure 2). Therefore, each MR image *I* undergoes an intensity non-uniformity correction using ANTs–N4BiasFieldCorrection (Tustison et al., 2010; Avants et al., 2011) to obtain the corrected image *I*^*Hom*^. This algorithm improves the N3 Intensity Inhomogeneity correction (Sled et al., 1998) and is based on the assumption that there exists a smooth, slowly varying multiplicative field *F* corrupting the image intensities: *I* = *I*^*Hom*^×*F*. In the specific case of early brain development where heterogeneous myelination occurs, the default correction algorithm might be insufficient and a dedicated correction method could be used following Prastawa et al. (2004) example. The choice of the most appropriate algorithm is let to the user. In any case, the Local Correlation Criteria (similar to ANTS Cross-correlation Avants et al., 2011) we use for the non-linear registration in Section 2.3.2 is robust to local intensity bias and is potentially able to cope with an incomplete inhomogeneity correction.

#### 2.1.4. Skull-stripping

It is often necessary (e.g., in Section 2.2.1) to process the brain without its surrounding skull. For this reason, the pipeline includes a skull-stripping step (also called non-brain removal tool). We selected Robex (Iglesias et al., 2011) for the robustness of its results with no parameter fine tuning: Iglesias et al. (2011) showed it generally performs better than six other popular algorithms (BET, Smith, 2002; BSE, Shattuck et al., 2001; FreeSurfer^3^; AFNI^4^; BridgeBurner, Mikheev et al., 2008; and GCUT, Mahapatra, 2012). Our experiments were in agreement with this affirmation: when using Robex on our datasets, we no longer had large parts of the skull remaining which was sometimes the case when using FSL–BET with the default parameters. Inputing *I*, the image with the brain and its surrounding skull, Robex outputs *I*^*robex*^ and *I*^*mask*^, the skull stripped brain and the corresponding region mask respectively. In fact, Robex applies an additional intensity inhomogeneity correction and thus modifies the intensity of the output image *I*^*robex*^. Therefore, one has to use the output mask *I*^*mask*^ and mask the original image *I* to obtain *I*^*brain*^, the image with the brain only (cf. Figure 2).

### 2.2. Position correction

Contrary to the previous section, the images are now treated depending on the subject (and time point). This module consists of two combined steps: (1) Longitudinal rigid registration, and (2) Affine spatial normalization. We first present these modules before explaining how we combine them.

#### 2.2.1. Longitudinal rigid registration

**Figure 3:**
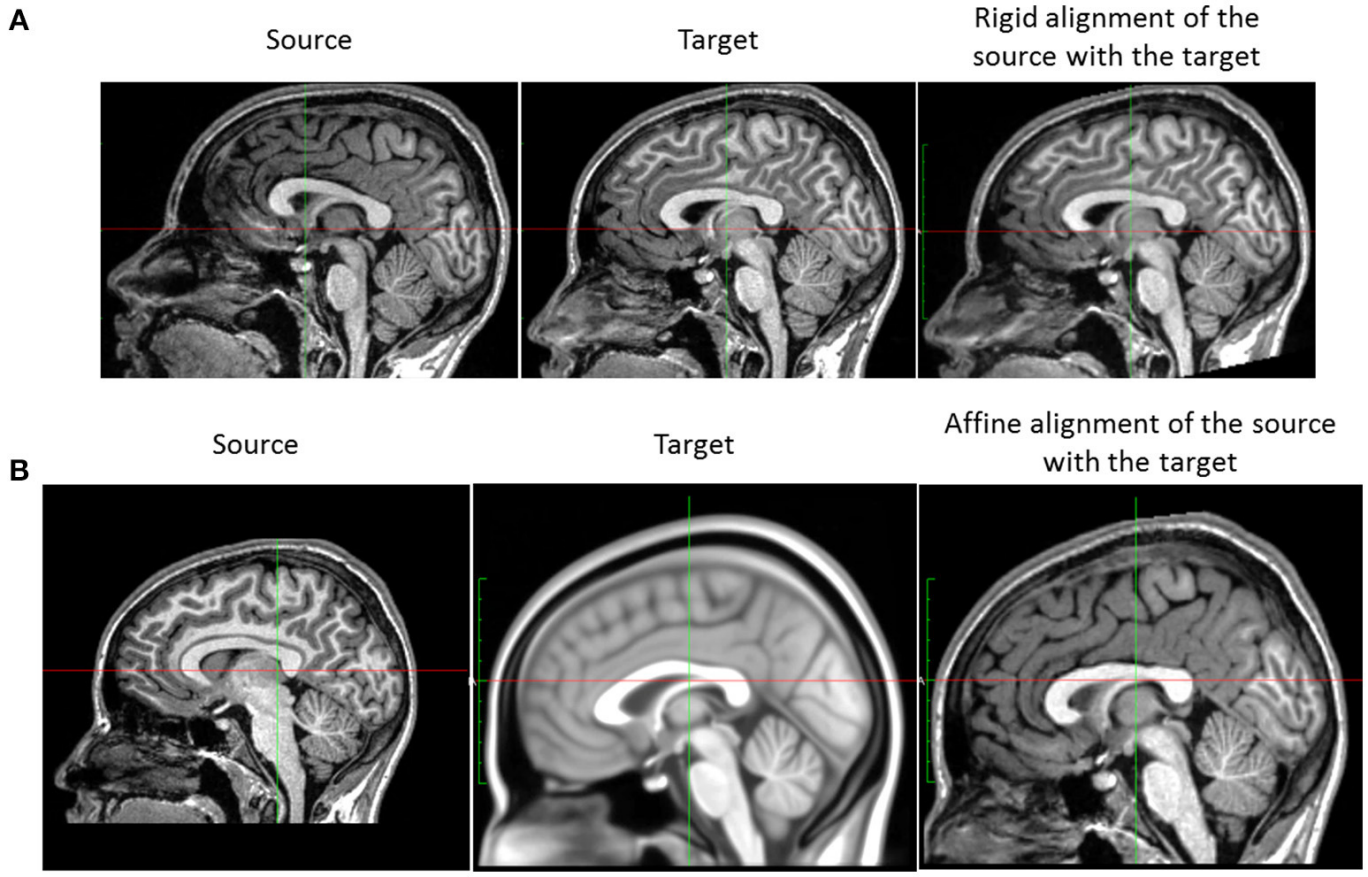
**Position correction steps**. **(A)** Rigid registration of subject images. The image on the left is the follow-up image of a subject, the baseline (used as the reference) being the image in the middle. The image on the right is the subject image after rigid alignment. **(B)** Affine normalization of a subject image. Left: subject image. Middle: the MNI152 template. The subject image and the template differ in size and orientation. Right: result of the affine normalization.

For a single subject, the acquisition at different time points is usually not performed with the same position of the head in the scanner. This creates a global rigid (six degrees of freedom) misalignment of each subject data series. Since the aim of this work is to model the subtle local longitudinal brain changes, we need to account for this source of variability that generally exceeds the longitudinal variability. Taking the baseline *I*_0_ as the reference position, we rigidly align the follow-up images *I*_1_,…, *I*_*N*−1_ to the baseline *I*_0_, using the rigid transformations $\phi_{R}^{1}$,…,$\phi_{R}^{N-1}$, to obtain the rigidly aligned image $I_{1}^{al}$,…, $I_{N-1}^{al}$ (cf. Figure 3).

We choose to use FSL–FLIRT (Jenkinson and Smith, 2001; Jenkinson et al., 2002) for the linear registration as it is the benchmark linear registration framework used in the influential work of Klein et al. (2009) for the comparison of several state-of-the-art non-linear registration algorithms. The different steps of the rigid registration step are described in Algorithm 1. We note that despite the optimization in two steps, only one single rigid transformation is applied. Composing the transformations from the—whole head and skull-stripped head—intra-subject rigid registrations minimizes the potential resampling artifacts introduced by the repeated resampling of the data (during the different rigid registration steps). Lastly, we use B-splines as the interpolation method (more accurate than the standard tri-linear interpolation, Parker et al., 1983) and the normalized correlation as the cost function.

**Algorithm 1:** Longitudinal Rigid Registration between 2 Images

**Input:** *I*_*j*_ with *j* = 1, …, *N*−1, the image not necessarily aligned with the reference *I*_0_.
**Output:** $I_{j}^{al}$ with *j* = 1, …, *N*−1, the image after rigid alignment with the reference *I*_0_.
Find the rigid transformation $\phi_{1}^{j}$ that aligns *I*_*j*_ to *I*_0_
$H_{j}=I_{j}\circ \phi_{1}^{j}$
Skull-strip (SS) *I*_0_ and *H*_*j*_
*J*_*j*_ = *SS*(*H*_*j*_) and *J*_0_ = *SS*(*I*_0_)
Find the rigid transformation $\phi_{2}^{j}$ that aligns *J*_*j*_ to *J*_0_
$K_{j}=J_{j}\circ \phi_{2}^{j}$
Compose the 2 previously found transformations
$\phi_{R}^{j}=\phi_{2}^{j}\circ \phi_{1}^{j}$
Apply the composed transformation to the input image *I*_1_
$I_{j}^{al}=I_{j}\circ \phi_{R}^{j}$

#### 2.2.2. Affine spatial normalization

Each brain differs in size and shape. In preparation for the group analysis and in order to align each subject anatomy in a common reference space, we normalize each subject head (shape and pose) to the MNI152 reference space using an affine (twelve degrees of freedom) transformation. Practically, the brain normalization consists in resampling each subject baseline image *I*_0_ in a common standard space *S*_*MNI*_ (MNI152 space) using an affine transformation ϕ_*A*_ computed with FSL–FLIRT to obtain the normalized image $I_{0}^{MNI}$ (see Figure 3). We use B-splines as the interpolation method and the normalized correlation as the cost function.

#### 2.2.3. Combined longitudinal rigid registration and spatial normalization

In the spirit of Section 2.2.1, we avoid as much as possible the potential resampling artifacts by composing the two spatial transformations ϕ_*R*_ and ϕ_*A*_ from the previous steps. The baseline *I*_0_, is spatially normalized to the MNI152 space using ϕ_*A*_ (cf. Section 2.2.2). Concerning the follow-up images *I*_*j*_, we apply the composition of ϕ_*A*_ and $\phi_{R}^{j}$ to *I*_*j*_. Since $I_{j}^{al}$ and *I*_0_ are already rigidly aligned the transformations that map both of them to the template *S*_*MNI*_ are the same.

### 2.3. Non-linear deformation analysis

After the correction of the images in position and intensity, we can estimate the residual longitudinal morphological differences using non-linear registration. For this non-linear registration step, all the subjects are processed independently in order to compute each individual longitudinal deformation (expressed in every subject anatomy but with the same coordinate space). The final step is done in three stages: (1) Estimation of the subject-specific longitudinal deformation trajectory using the previously computed longitudinal deformations, (2) Study-specific template creation, and (3) Transport of the subject-specific longitudinal deformation trajectory in the template (cf. **Figure 6**). Before going further, we introduce the mathematical formalism related to Deformation Based Morphometry.

#### 2.3.1. Mathematical formalism for deformation-based morphometry

The longitudinal evolution of a point *x* of the brain between the initial biological time point *t*_0_ = 0 and the biological time *t*_1_ is defined by the *deformation* ϕ that maps the initial position *x*(*t*_0_) to the position *x*(*t*_1_):

$$\phi :{\mathbb{R}}^{n}\times \mathbb{R}\to {\mathbb{R}}^{n}$$

(x,t)↦x(t)=ϕ(x,t)

In neuroimaging, the preservation of the brain topology is important; it can be obtained under the large deformation diffeomorphic setting (Joshi and Miller, 2000; Beg et al., 2005). In this framework, we define the transformations φ that belong to the group G of diffeomorphisms: differentiable bijections with differentiable inverse. The transformations are parameterized by the flow of time-dependent velocity vector fields *v*(*x, s*) (with the parametrization time *s* ∈ [0, 1]) specified by the following ordinary differential equation:

$$\frac{\partial \varphi \left(x,s\right)}{\partial s}=v\left(\varphi \left(x,s\right),s\right),$$

with φ(*x*, 0) = *Id*(*x*) (identity transformation). The resulting deformation ϕ, mapping *x*(*t*_0_) to *x*(*t*_1_) is given by the flow at *s* = 1: ϕ(*x, t*_1_) = φ(*x*, 1). In the spirit of the log-Euclidean framework, Arsigny et al. (2006) proposed to restrict to the one-parameter subgroup of diffeomorphisms where the velocity vectors are stationary (i.e., constant over the parametrization time s): *v*(*x, s*) = *v*(*x*). In this case, the transformation ϕ(*x, t*_1_) is encoded by the *stationary velocity field* (SVF) *v*(*x*) via the Lie group exponential map: ϕ(*x, t*_1_) = exp(*v*(*x*)); the exponential map is defined as the flow of the stationary ordinary differential equation:

$$\frac{\partial \varphi \left(x,s\right)}{\partial s}=v\left(\varphi \left(x,s\right)\right),$$

with φ(*x*, 0) = *Id*(*x*) and *s*∈[0, 1].

#### 2.3.2. Non-linear symmetric diffeomorphic registration with confidence mask

We estimate the subtle longitudinal changes using symmetric non-linear diffeomorphic registration. The diffeomorphic deformations are parameterized using Stationary Velocity Fields (SVF), providing us with a rich mathematical and computational setting (see Arsigny et al., 2006; Vercauteren et al., 2008; Lorenzi et al., 2011).

**Figure 4:**
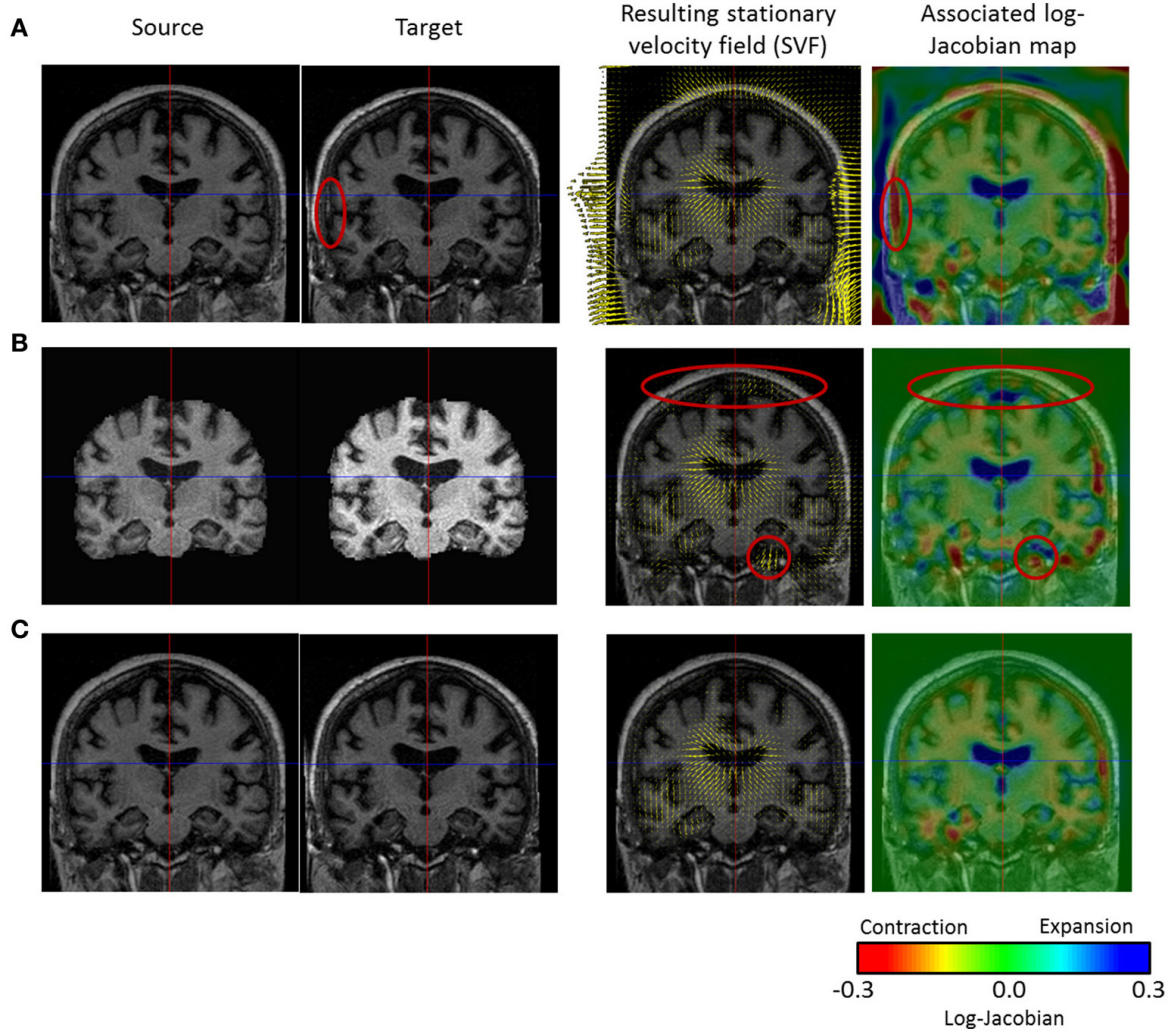
**Comparison of three non-linear diffeomorphic registration methods**. First and second column: we see the intensity bias affecting the source and target images. **(A)** Registration of the head with no confidence mask: strong deformation fields are estimated in the skull and meninges that diffuse to the outer cortex region and bias the results (cf. red ellipse where a non-realistic expansion of 38% is found). **(B)** Registration of the skull-stripped images (no confidence mask): the use of the skull-stripped images biases the result at the level of the outer cortex (cf. red ellipses) where non-existing high value deformations are found due to the high intensity gradient. In fact, skull-stripping imposes the outside brain intensity to be zero creating a high intensity gradient that biases the registration results (the update δ*v*_*i-j*_ is directly proportional to the image gradient). **(C)** Registration of the head with confidence mask: the registration using the confidence mask enables us to estimate realistic transformations in the outer cortex.

To non-linearly register *I*_*i*_ to *I*_*j*_, we estimate the Stationary Velocity Field *v*_*i-j*_ (cf. Figure 4) via an alternate minimization of the following log-Demons energy with respect to *v*_*i-j*_ and the auxiliary SVF *v*_*c*_ (Cachier et al., 2003). Instead of minimizing a global energy, a correspondence field *v*_*c*_ is introduced, so that two simple, fast, and more efficient minimization steps are performed, respectively for *E*_*Sim*_ and *E*_*Reg*_. In the first step, *E*_*Sim*_ is minimized using a gradient descent method, whereas in the second step *E*_*Reg*_ can be solved explicitly as the Gaussian convolution of *v*_*c*_ when the regularization term is chosen adequately: 
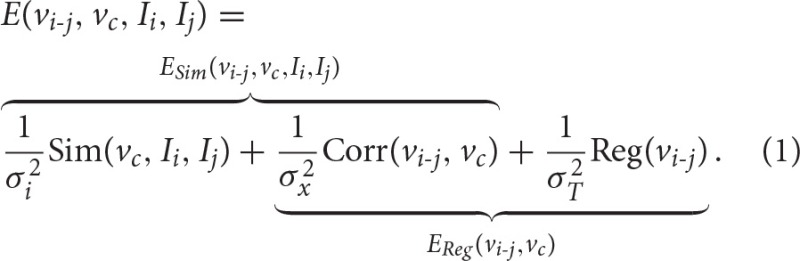
 In this formula, σ_*i*_ is the parameter linked to the noise in the image, σ_*x*_ is linked to the uncertainty of the matching in the correspondence term, σ_*T*_ is the regularization weight, **Sim** is the similarity criterion, **Reg** the regularization term, and **Corr** is the correspondence term that links *v*_*i-j*_ to *v*_*c*_. The LCC log-Demons (Lorenzi et al., 2013) uses ρ the Local Correlation Coefficient (LCC) similarity metric (Cachier et al., 2003) since it is robust to local intensity artifacts:

$$\rho (I_{i},I_{j})=\int_{\Omega }\frac{\bar{I_{i}I_{j}}}{\sqrt{\bar{I_{i}^{2}}\bar{I_{j}^{2}}}}\text{ with }\bar{I}=G_{\sigma }*I,$$

where *G*_σ_ is the Gaussian smoothing operator with a kernel size of σ and Ω is the image domain.

Therefore, by considering the symmetric resampling $I_{i}^{\prime }=I_{i}\circ \exp\left(\frac{v_{c}}{2}\right)$ and $I_{j}^{\prime }=I_{j}\circ \exp\left(-\frac{v_{c}}{2}\right)$, the first term of Equation (1) can be written as:

$$\begin{array}{l} \text{Sim}\left(v_{c},I_{i},I_{j}\right)=\rho^{2}\left(I_{i}^{\prime },I_{j}^{\prime }\right)=\rho^{\prime 2}\left(v_{c},I_{i},I_{j}\right)\\ \;=\frac{{\left[\bar{I_{i}\circ \exp\left(\frac{v_{c}}{2}\right).I_{j}\circ \exp\left(-\frac{v_{c}}{2}\right)}\right]}^{2}}{\bar{{\left[I_{i}\circ \exp\left(\frac{v_{c}}{2}\right)\right]}^{2}}.\bar{{\left[I_{j}\circ \exp\left(-\frac{v_{c}}{2}\right)\right]}^{2}}}\text{.} \end{array}$$

If we define the update field δ*v*_*i-j*_ through the zeroth order term of the Baker-Campbell-Hausdorff (BCH) formula (Bossa et al., 2007):

$$\delta v_{i\text{-}j}=\log\left(\exp\left(-v_{i\text{-}j}\right)\circ \exp\left(v_{c}\right)\right)\approx -v_{i\text{-}j}+v_{c}\text{,}$$

then in the first part of the alternate optimization of Equation (1), *E*_*Sim*_ has to be minimized with respect to δ*v*_*i-j*_ :

$$E_{Sim}\left(\delta v_{i\text{-}j},I_{i},I_{j}\right)=-\frac{1}{\sigma_{i}^{2}}\rho^{\prime 2}\left(\delta v_{i\text{-}j},I_{i},I_{j}\right)+\frac{1}{\sigma_{x}^{2}}||\delta v_{i\text{-}j}|{|}^{2}\text{,}$$

with $\text{Corr}\left(v_{i\text{-}j},v_{c}\right)=||\log\left(\exp\left(-v_{i\text{-}j}\right)\circ \exp\left(v_{c}\right)\right)|{|}^{2}=||\delta v_{i\text{-}j}|{|}^{2}$.

In the second part of the optimization, *E*_*Reg*_ should be minimized with respect to *v*_*i-j*_ :

$$E_{Reg}\left(v_{i\text{-}j},v_{c}\right)=\frac{1}{\sigma_{x}^{2}}||\log\left(\exp\left(-v_{i\text{-}j}\right)\circ \exp\left(v_{c}\right)\right)|{|}^{2}+\frac{1}{\sigma_{T}^{2}}Reg\left(v_{i\text{-}j}\right)\text{.}$$

The registered images generally comprise the brain and its surrounding skull which can lead to corrupted results. In fact, the resulting deformation field generally exhibits high values in the region of the meninges and the skull that diffuse through regularization in the outer cortex (see Figure 4), potentially yielding to misleading discoveries.

One solution is to only register the brain tissues and the cerebrospinal fluid (CSF) obtained through skull-stripping. However, this solution may be prone to errors (small parts of the outer cortex could be cropped) and puts the outside brain intensity to zero creating a high intensity gradient that biases the registration results (as shown on Figure 4), since the update δ*v*_*i-j*_ is directly proportional to the image gradient.

Therefore, we modified the LCC log-Demons algorithm to incorporate the use of a confidence mask as proposed by Brett et al. (2001), and first introduced in the Demons algorithm by Stefanescu et al. (2004). We consider that we do not want to align the structures outside the brain (skull, meninges,…). Therefore, the voxels outside the brain should have no influence in the similarity minimization step. We define a probabilistic mask ω(*x*) such that its value is ω(*x*) = 1 for a voxel inside the brain, ω(*x*) = 0 outside, and in-between depending on the confidence we have for the voxel. The new log-Demons energy to minimize is:

$$\begin{array}{l} E\left(v_{i\text{-}j},v_{c},I_{i},I_{j}\right)=\omega \frac{1}{\sigma_{i}^{2}}\text{Sim}\left(v_{c},I_{i},I_{j}\right)\;+\;\frac{1}{\sigma_{x}^{2}}\text{Corr}\left(v_{i\text{-}j},v_{c}\right)\\ \;+\frac{1}{\sigma_{T}^{2}}\text{Reg}\left(v_{i\text{-}j}\right)\text{.} \end{array}$$

Thus, only the first part of the minimization (*E*_*Sim*_) is modified and we still get a closed-form solution leading to an effective computational scheme for the optimization of *E*_*Sim*_ [cf. demonstration in Appendix (Supplementary Materials)]:

$$\delta v_{i\text{-}j}=\{\begin{array}{l} -\frac{2\Lambda }{||\Lambda |{|}^{2}+\frac{1}{\omega }\frac{4}{\rho^{2}}\frac{\sigma_{i}^{2}}{\sigma_{x}^{2}}}\text{, if }\omega >0\\ 0,\text{ if}\omega =0 \end{array}$$

with

(2) $$\begin{array}{r} \Lambda =\frac{G_{\sigma }*\left(I_{i}\nabla I_{j}^{T}\right)}{G_{\sigma }*\left(I_{i}I_{j}\right)}-\frac{G_{\sigma }*\left(I_{j}\nabla I_{i}^{T}\right)}{G_{\sigma }*\left(I_{i}I_{j}\right)}+\frac{G_{\sigma }*\left(I_{i}\nabla I_{i}^{T}\right)}{G_{\sigma }*\left(I_{i}^{2}\right)}\\ \;-\frac{G_{\sigma }*\left(I_{j}\nabla I_{j}^{T}\right)}{G_{\sigma }*\left(I_{j}^{2}\right)}\text{.} \end{array}$$

In order to keep a symmetric formulation of the registration, the probabilistic mask ω is defined using two masks. The first one is the brain mask *M* of the moving image and the second one is the brain mask *F* of the fixed image. The mask ω is then defined as the average of the symmetric resampling of the two brain masks in the halfway space:

$$\omega =\frac{1}{2}\left[M\circ \exp\left(\frac{v_{c}}{2}\right)+F\circ \exp\left(-\frac{v_{c}}{2}\right)\right]$$

Hence, the registration problem is still defined on the whole image domain but the update is weighted differently depending on the confidence on the brain areas. In our experiments, we defined the initial brain masks (for both fixed and moving images) as binary masks.

#### 2.3.3. Estimation of the subject-specific longitudinal trajectory via fully symmetric SVF regression

Given the previously estimated series of longitudinal deformations ϕ_*i-j*_ = exp(*v*_*i-j*_) with 0 ≤ *i* < *j* ≤ *N* − 1 for a subject, we then model the *subject-specific longitudinal deformation trajectory* $\hat{\phi }$ as :

$$\hat{\phi }\left(x,t\right)=\exp\left(t\cdot \hat{v}\left(x\right)\right)\text{with}t\in \mathbb{R}\text{,}$$

where $\hat{v}$ is the best fit of a fully symmetric linear model in time—through the origin—of the series of SVFs *v*_*i-j*_ :

$$\begin{array}{l} \hat{v}=\underset{v}{\text{arg min}}\sum_{0\leq i<j\leq N-1}\|(t_{j}-t_{i})v-v_{i\text{-}j}{\|}^{2}\\ \;=\frac{\sum_{0\leq i<j\leq N-1}(t_{j}-t_{i})v_{i-j}}{\sum_{0\leq i<j\leq N-1}{\left(t_{j}-t_{i}\right)}^{2}}\text{.} \end{array}$$

This model uses all the possible combinations of SVFs *v*_*i-j*_ between the different time points while using the symmetry of the pairwise registration (*v*_*i-j*_ = −*v*_*j-i*_) to simplify the problem. $\hat{v}$ and $\hat{\phi }\left(t=1\right)=\exp\left(\hat{v}\right)$ represent the subject-specific evolution trajectory over a year. One should note that a linear model of the longitudinal SVFs does not lead to a linear model of the deformations. For up to three time points, our experience showed that a linear model in time is sufficient to explain the data. A higher-order model could be used for a higher number of time points at the cost of increasing the statistical complexity.

#### 2.3.4. Unbiased study-specific template construction

**Figure 5:**
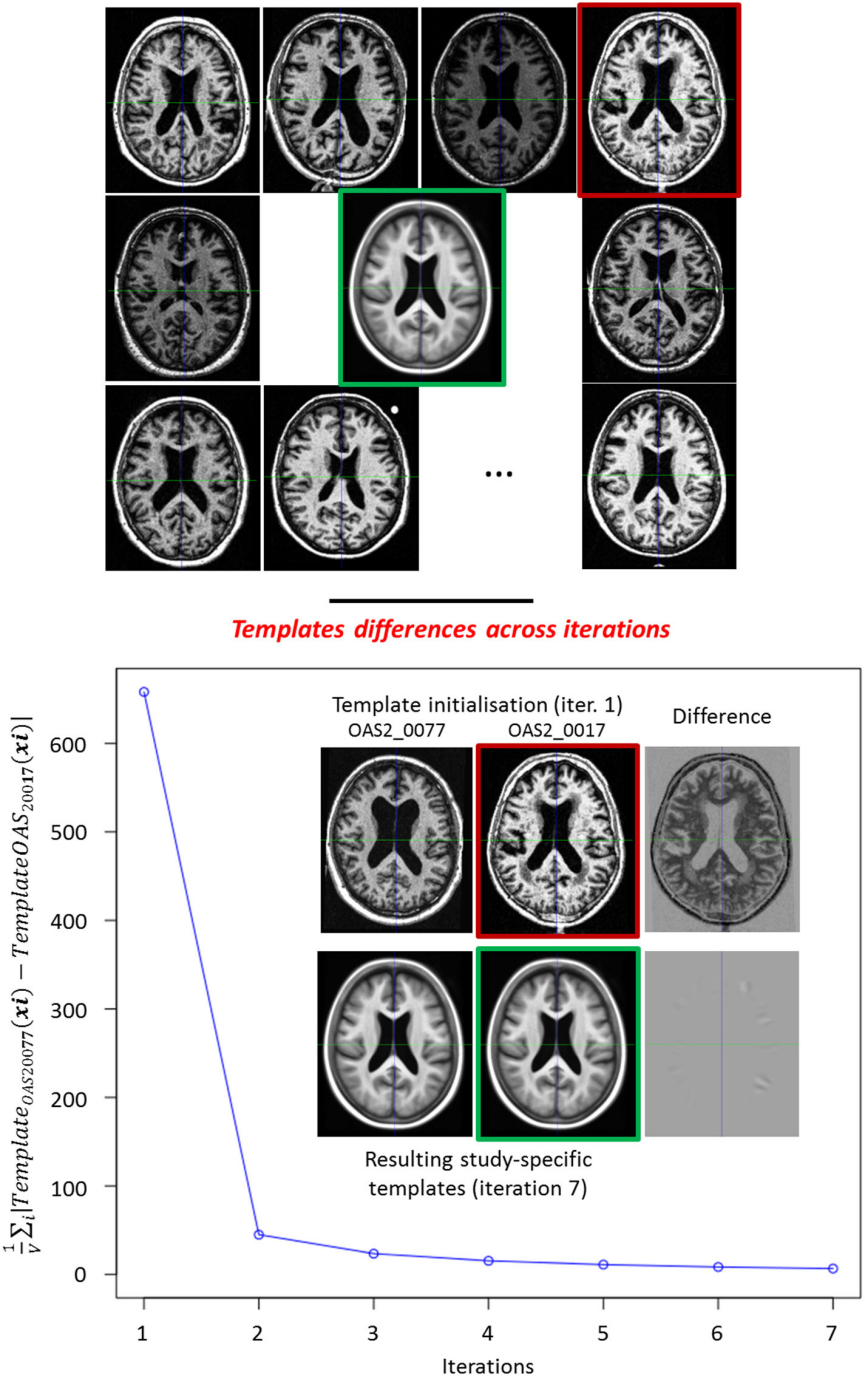
**Top:** Iterative template Construction. Example of the construction of the template (green frame) of a study of 136 subjects, 9 subjects are displayed. Red frame: the reference subject (OAS2_0017) used for the initialization. **Bottom:** Influence of the reference subject used to initialize the study-specific template. We built a second study-specific template by initializing it with a different subject (OAS2_0077). We computed $\frac{1}{V}\underset{i}{\sum }|Template_{OAS2\text{\_}0077}\left(x_{i}\right)-Template_{OAS2\text{\_}0017}\left(x_{i}\right)|$ over the brain mask at each iteration. Although the initial reference images are dissimilar, we obtain two very similar templates at the end.

In order to compare all the subject-specific longitudinal deformation trajectories, we need to have these deformations normalized in the same common reference anatomy called study-specific template *A*. Although each subject brain is normalized to the standard space (cf. Section 2.2.2), the affine alignment is not sufficient to compensate for the local anatomical differences (there is no voxel-to-voxel correspondence yet between the different anatomies). Among the available methods for the template construction, we chose to use the method from Guimond et al. (2000) consisting in the iterative averaging of intensities and deformations. This iterative process is described in Algorithm 2 and illustrated on 136 subjects (Figure 5). In the following experiments, the iterative algorithm was stopped at the seventh iteration. At a given iteration there are two successive image resamplings due to the application of two deformations; this can bias the centering of the template. To ensure it is centered, we minimize the number of image resamplings at a given iteration by using a zeroth order term of the BCH: $\log\left(\exp\left(v_{k}^{i}\right)\circ \exp\left(-{\overset{-}{v}}_{k}\right)\right)\approx v_{k}^{i}-{\overset{-}{v}}_{k}$. Moreover, a good practice for the selection of the initialization image for *A*_0_ is to manually choose a subject image that is roughly centered with respect to the considered sample in order to avoid being blocked in a local minimum. In practice, we checked that changing the reference image for *A*_0_ changed the final template *A* by only a negligible amount as shown on Figure 5.

**Algorithm 2:** Creation of an Unbiased Template *A*

**Input:** Set of study images *I*^*i*^
**Output:** *A*: Study-specific template image
Initialization: Select a reference image *I*^*j*^ among the M subjects images
$A_{0}=I^{j}$
**repeat**
Non-linearly register the images to *A*_*k*_
$A_{k}\approx I^{i}\circ \exp\left(v_{k}^{i}\right)$
Mean stationary velocity field
${\overset{-}{v}}_{k}=\frac{1}{M}\left(\overset{M}{\underset{i=1}{\sum }}v_{k}^{i}\right)$
Resample subjects' image
$L_{k}^{i}=I^{i}\circ \exp\left(v_{k}^{i}-{\overset{-}{v}}_{k}\right)$
Template iteration k+1: Mean intensity image
$A_{k+1}=\frac{1}{M}\left(\overset{M}{\underset{i=1}{\sum }}L_{k}^{i}\right)$
**until** Variations of *A*_*k*_ and ${\overset{-}{v}}_{k}$ are very small:
$\frac{1}{V}\overset{V}{\underset{i=1}{\sum }}{\left(A_{k+1}\left(i\right)-A_{k}\left(i\right)\right)}^{2}$ and $||{\overset{-}{v}}_{k+1}-{\overset{-}{v}}_{k}||<\epsilon$
*A* = *A*_*k*+1_

Here again the non-linear registrations are performed using our modified LCC log-Demons algorithm with confidence mask (we used the subjects images masks), in order to estimate the study-specific template while being robust to the artifacts on the brain boundaries.

Another point concerns the choice of the time point at which the template is created. There is no golden rule and the choice of the time point is usually let to the user. As for us, we use the images *I*_0_ at the first time point *t*_0_ to create the template.

#### 2.3.5. Parallel transport of the subject-specific longitudinal stationary velocity field

**Figure 6:**
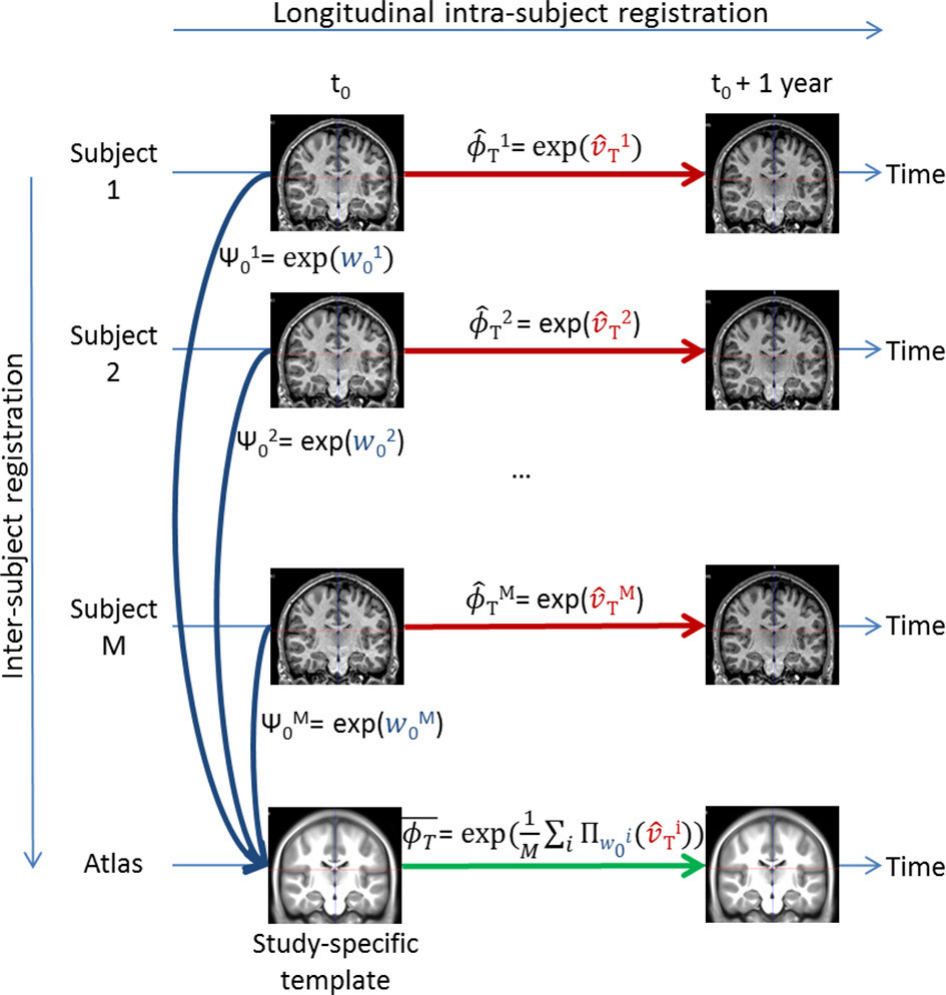
**Illustration of the parallel transport of the study transformations to the study-specific template**. After each subject longitudinal SVF transport, the mean transformation ${\overset{-}{\phi }}_{T}$ is computed by taking the exponential of the average of all the transported subject-specific longitudinal SVFs $\Pi_{w_{0}^{i}}\left({\hat{v}}_{T}^{i}\right)$.

Now that a common brain anatomical image is defined, we need to express each subject-specific longitudinal deformation trajectory $\hat{\phi }$ in the template anatomy to be able to compare them. To do so, we use the parallel transport computed with the Pole ladder (Lorenzi and Pennec, 2013) of the subject-specific longitudinal SVF trajectory $\hat{v}$ along the inter-subject SVF *w*_0_ parameterizing the cross-sectional transformation ψ_0_ = exp(*w*_0_) that maps *I*_0_ to *A* (cf. Figure 6).

The result is ${\hat{v}}_{T}=\Pi_{w_{0}}\left(\hat{v}\right)$, the subject-specific longitudinal SVF trajectory normalized in the template space. We can then compute the subject-specific longitudinal deformation trajectory in the template space ${\hat{\phi }}_{T}=exp\left({\hat{v}}_{T}\right)$. The different steps necessary for the parallel transport are described in Algorithm 3. It is then possible to perform a statistical analysis on these transported subject-specific longitudinal stationary velocity fields ${\hat{v}}_{T}$ as shown in Section 3.

**Algorithm 3:** Pole Ladder for the Parallel Transport of the Longitudinal Stationary Velocity Field

**Input:** $\hat{v}$ : subject-specific SVF, *I*_0_: subject image where the SVF is normalized and the template *A*
**Output:** ${\hat{v}}_{T}$ : subject transported SVF
Non-linearly register the subject image *I*_0_ into *A*
*A* ≈ *I*_0_ ∘ exp(*w*_0_)
Parallel transport of $\hat{v}$ along *w*_0_
Scaling step: find *n* such that *w*_0_∕*n* is smaller than 0.5 voxel in all dimensions
$n=ceiling(\frac{\max_{x\in \Omega }||w_{0}(x)||}{0.5\cdot voxelsize})$
**repeat**
Ladder step:
$v_{k}=\hat{v}+\left[\frac{w_{0}}{n},\hat{v}\right]+\frac{1}{2}\left[\frac{w_{0}}{n},\left[\frac{w_{0}}{n},\hat{v}\right]\right]$ with [ , ] the Lie brackets:
$$\begin{array}{l} \left[v,w\right]\left(x\right)=D_{w}v\left(x\right)-D_{v}w\left(x\right)\\ =\underset{i}{\sum }\left(w_{i}\left(x\right)\frac{\partial v_{i}\left(x\right)}{\partial x_{i}}-v_{i}\left(x\right)\frac{\partial w_{i}\left(x\right)}{\partial x_{i}}\right) \end{array}$$
where *v*_*i*_(*x*) and *w*_*i*_(*x*) are respectively the components of the vector fields *v*(*x*) and *w*(*x*) in a Cartesian coordinates system of the point *x* with coordinates *x*_*i*_. The numerical computation of the derivatives is performed using a centered difference scheme.
Let $\hat{v}=v_{k}$
**until** *k* = *n*
${\hat{v}}_{T}=\Pi_{w_{0}}\left(\hat{v}\right)=v_{n}$

## 3. Application to the analysis of the longitudinal changes in Alzheimer's disease

The aim of this section is to show an application of the proposed processing pipeline. We focused our illustration on Alzheimer's disease, a neuro-degenerative disease that causes dramatic changes in the brain anatomy over time. We use the OASIS database (Marcus et al., 2010).

### 3.1. OASIS database

The clinical cohort considered in this study is composed of 64 patients diagnosed with very mild to moderate Alzheimer's disease, and 72 healthy individuals. For these subjects, 2 to 5 longitudinal brain acquisitions (T1 Magnetic Resonance Imaging) were available, corresponding to a follow-up time *t*_0*-j*_ = *t*_*j*_−*t*_0_ of 0.5 to 6.9 years. Further information can be found in Appendix (Supplementary Materials).

### 3.2. Methods and results

After applying the processing pipeline to the database (the parameters used for the different steps are summarized Table 1), we obtain the transported subject-specific longitudinal deformation trajectories ${\hat{\phi }}_{T}^{i}\left(t\right)=\exp\left(t\cdot {\hat{v}}_{T}^{i}\right)$ for each subject *i* in the study-specific template: we thus get 72 subject-specific longitudinal SVFs ${\hat{v}}_{T}^{i}$ for the healthy controls and 64 for the patients with Alzheimer's disease.

**Table 1:** **Parameters used for each module of the longitudinal study**.

**Pipeline step**	**Parameters values**
Standard reorientation	*Default*
Field of view reduction	*Default*
Intensity inhomogeneity corr.	*Default*
Skull-stripping	*Default*
Longitudinal rigid registration	−cost normcorr -interp spline -dof 6
Affine registration	−cost normcorr -interp spline
Non-linear reg.: Intra- and Inter-subject	−r 2 -R 1 -C 3 -a 30x20x10 -x 0 -b 2.0 -S 0.15 -u 3.0 -V
Transport	*Default*

Concerning the non-linear registration parameters for the LCC log-Demons, the optimal parameters we propose here would of course be different for another study, and we recommend to fine-tune in priority the amount of regularization (-b) and the number of iterations (-a). As for the SVF exponentiation (and log-Jacobian), all the computations were performed using an Euler forward integration scheme (option -z 1 in SVFLogJacobian tool).

**Figure 7:**
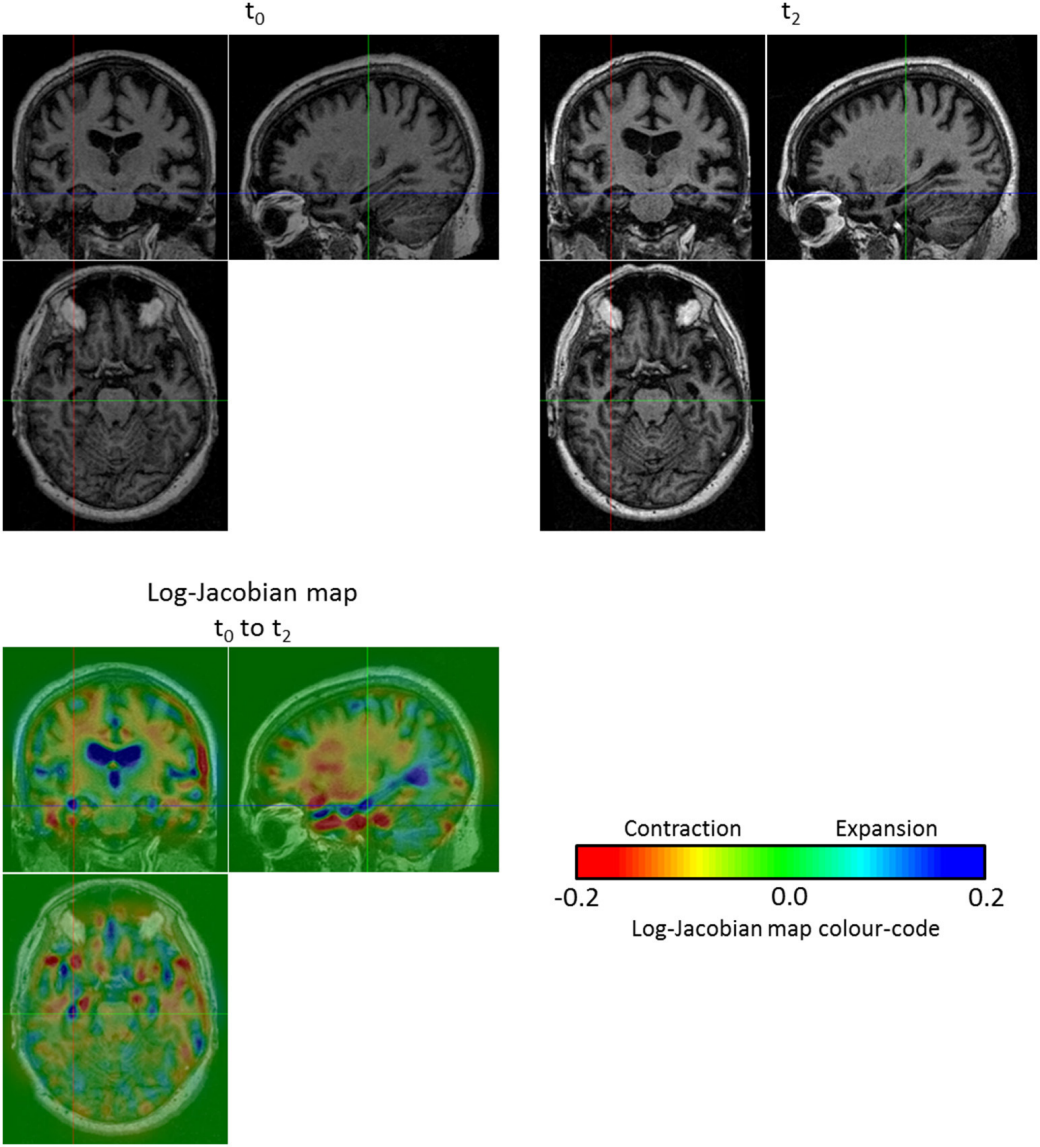
**Log-Jacobian map for the subject OAS2_0002**. We computed the log-Jacobian map—which represents the relative change of volume—for the SVF of the longitudinal evolution between *t*_0_ and *t*_2_. We can observe an expansion in the ventricles and more particularly in the temporal horn of the lateral ventricles and a contraction in the hippocampi. Moreover, although there is an artifact outside the brain (left hand edge of the follow-up image at *t*_2_), the use of the non-linear registration with confidence mask enables us to avoid any artifactual volume change in our log-Jacobian map.

Before discussing the results of the group-wise comparisons of the longitudinal evolutions, let us focus on an illustrative result concerning a single subject (OAS2_0002). We computed the log-Jacobian map—which quantifies the relative volume changes associated to the longitudinal deformation—for the SVF *v*_0-2_ of the longitudinal evolution between *t*_0_ and *t*_2_; the result can be seen on Figure 7. We can observe the expansion of the ventricles and more particularly in the temporal horn of the lateral ventricles, as well as the contraction in the hippocampi. Moreover, there exists an artifact outside the brain (left hand edge of the follow-up image on Figure 7). The use of the non-linear registration with confidence mask enables us to avoid any artifactual volume change in our log-Jacobian map and therefore provides more stable results. This is illustrated in Figure 4, where we compare the deformation found with and without the use of the confidence mask; we see on the left hand of the image (red circle on image A.) that this kind of artifact can locally bias the estimation of longitudinal deformations when the mask is not explicitly accounted for (Ashburner and Ridgway, 2013).

**Figure 8:**
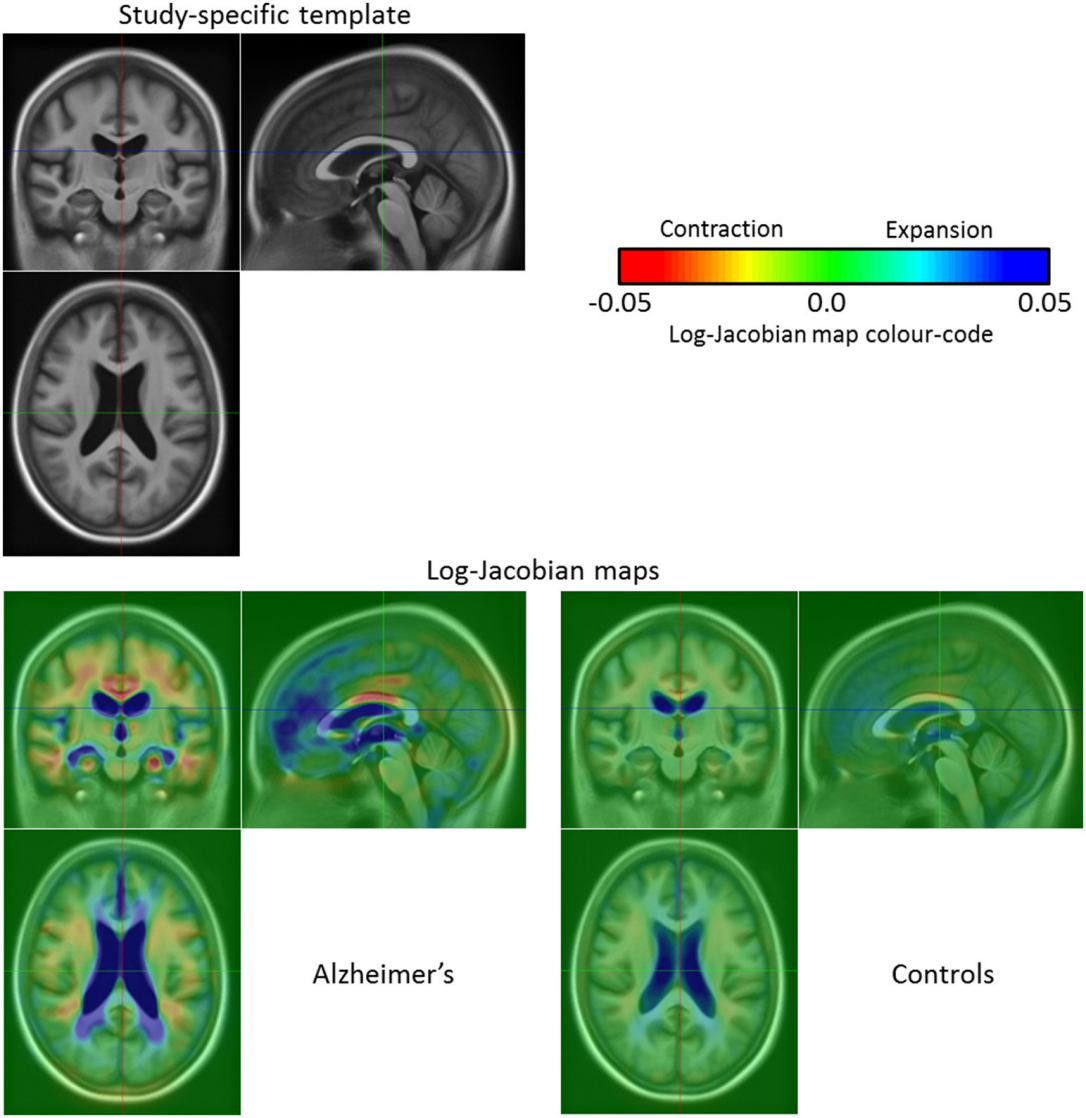
**Template for the 136 OASIS subjects at ***t***_**0**_ and log-Jacobian maps (1 year evolution) of the patients with Alzheimer's disease and the healthy control group**. The main expansion region concerns the lateral ventricles where the Alzheimer's patients exhibit higher values when compared to the healthy subjects. Moreover, for the patients with Alzheimer's disease we can see an expansion in the temporal horn of the lateral ventricles that does not exist in the non-demented control group. Finally, the atrophy is higher for the Alzheimer's patients and mainly located in several parts of the white matter, in the thalamus and in the hippocampi whereas there is no visible contraction in the thalamus or the hippocampi for the healthy group.

Concerning the groups study, we consider the subject-specific deformations over a year (*t* = 1) so that we study the SVFs ${\hat{v}}_{T}^{i}$. It is then possible to visualize the mean volume changes during 1 year for each group of patients with Alzheimer's disease and healthy controls. After computing the average SVF for the non-demented group and the Alzheimer's one, we compute the associated log-Jacobian maps^5^ (cf. Figure 8), and compare the modeled group-wise evolutions. We can see that the main expansion region is located in the lateral ventricles with higher values for the Alzheimer's patients group than for the healthy control one. Moreover, for the patients with Alzheimer's disease we can see an expansion in the temporal horn of the lateral ventricles that does not exist in the control group. Finally, the atrophy is higher for the Alzheimer's patients and mainly located in several parts of the white matter, in the thalamus and in the hippocampi whereas there is no visible contraction in the hippocampi or in the thalamus for the control group. These results are coherent with the findings reported in the literature (Braak and Braak, 1991; Fox et al., 1996; Jack et al., 2004; Schott et al., 2005; de Jong et al., 2008).

#### 3.2.1. Two-sample *t*-test: Alzheimer's patients vs. healthy controls

**Figure 9:**
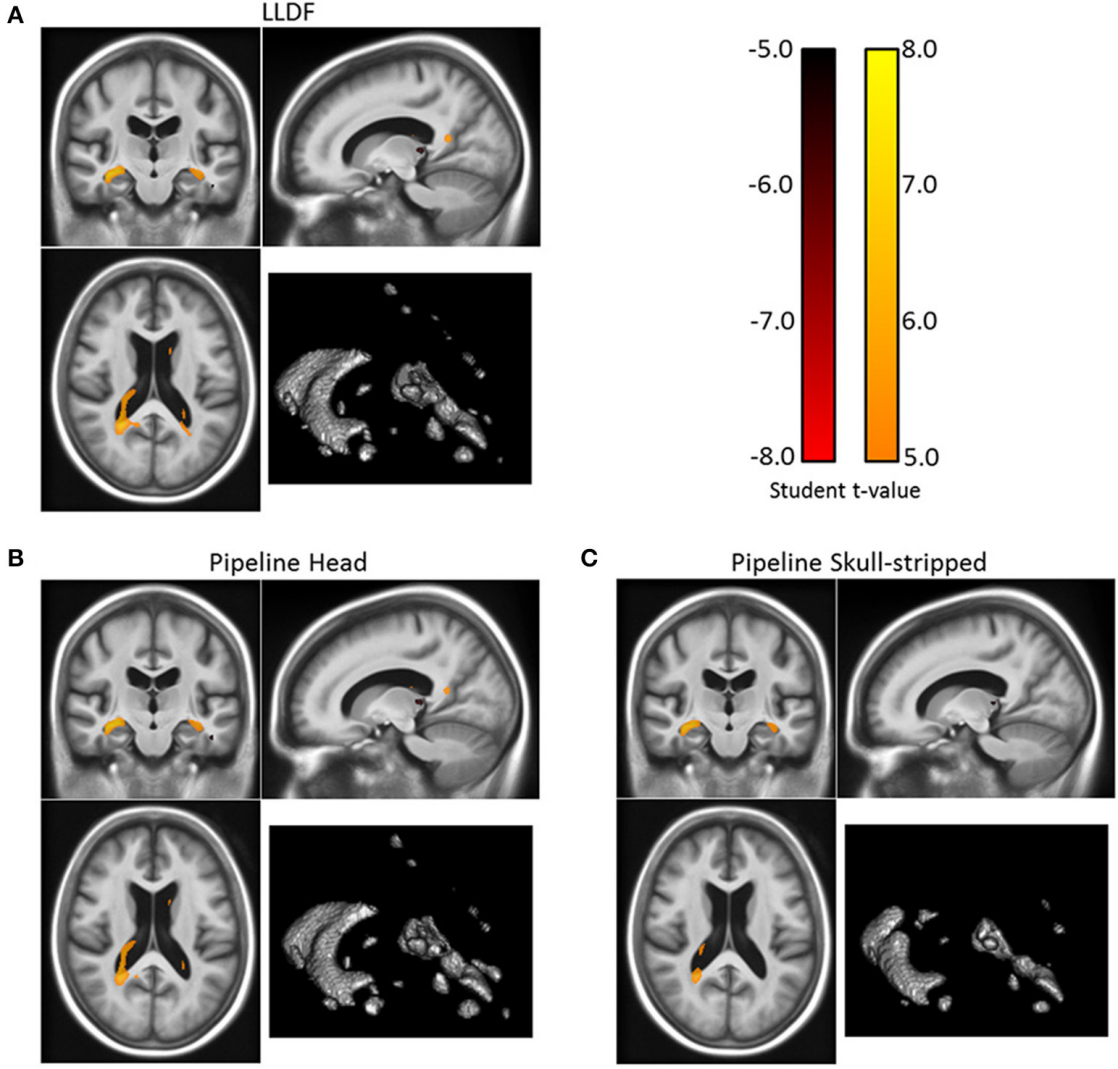
**Corrected t-statistic map for the volume changes differences between the patients with Alzheimer's disease and the healthy control group (for the 3 registration methods) on one slice**. The three results present similar patterns with statistical differences in the ventricular region, more particularly in the temporal horn of the lateral ventricles, and also in the thalamus. The volume of the regions of statistical significant differences are 10.4, 16.5, and 17.5 cm^3^ for respectively “Pipeline Skull-stripped” **(C)**, “Pipeline Head” **(B)**, and “LLDF” **(A)**. Moreover, the *t*-values are higher with the “LLDF” than with the two other methods. (Correction for multiple testing using the Family-Wise Error rate with a corrected *p*-value of 0.05).

We now statistically investigate the group-wise differences between the modeled longitudinal evolutions of the Alzheimer's patients group and the healthy control group by using a voxel-wise two-sample *t*-test on the log-Jacobian maps. For illustrative purposes, we show here a standard univariate analysis on a scalar map, but the use of the parallel transport in our pipeline enables us to do statistics directly on the subject-specific SVFs as shown in Section 3.2.3. The null hypothesis is that there exists no difference between the mean of the two groups. We used SPM8 (see Friston, 2007) for this test and corrected for multiple testing using the Family-Wise Error rate (FWE) with a corrected *p*-value of 0.05 in order to control for the same level of specificity. The *t*-test was limited to the brain mask. The result map with the thresholded *t*-values can be seen on Figure 9. The statistically different volume changes occur in the lateral ventricles, more particularly in the temporal horn, and also in the thalamus.

#### 3.2.2. Reliability of the LCC log-demons with a confidence mask

We tested the reliability of the implemented LCC log-Demons registration with a confidence mask. We compared it with the original LCC log-Demons applied to full head images or skull-stripped images. We therefore ran three similar processing pipelines where the only difference was the non-linear registration method used; the processing pipeline using the LCC log-Demons with a confidence mask is denoted as **LLDF**, the one using the registration of the whole head is called **Pipeline Head**, and the pipeline registering skull-stripped images is denoted as **Pipeline Skull-stripped**. Similarly to Section 3.2.1, we investigated the differences between the Alzheimer's patients group and the healthy control group in each case and compared the obtained results to see which method has the highest statistical sensitivity to find volume changes between the two groups.

The three corrected t-maps are presented Figure 9,^6^. The three results present similar patterns with most of the statistical differences in the ventricular region and more particularly in the temporal horn of the lateral ventricles. Other statistical differences can be found in the thalamus. The volume of the regions of statistical significant differences are 10.4, 16.5, and 17.5 cm^3^ for respectively “Pipeline Skull-stripped”, “Pipeline Head”, and “LLDF”. Moreover, the *t*-values are higher with the “LLDF” than with the two other methods. In average on the same statistical region (the smallest region, obtained by computing the intersection of the three statistically significant regions), we obtain an absolute *t*-value of 6.13 with “LLDF” against 5.98 with “Pipeline Head”, and 5.69 with “Pipeline Skull-stripped”. This increase of the *t*-values can be explained by the increased group difference for “LLDF” compared to the group differences of the other two methods and not by a reduction of the variance. On the same statistical region, we observe a relative increase of 23.4% with respect to “Pipeline Head” and of 23.7% with respect to “Pipeline Skull-stripped”. Therefore, the LLDF pipeline enables us to have an increased statistical sensitivity with no decrease of the specificity.

#### 3.2.3. Illustration of a DBM analysis: Hoteling's two-sample *T*^2^-test

**Figure 10:**
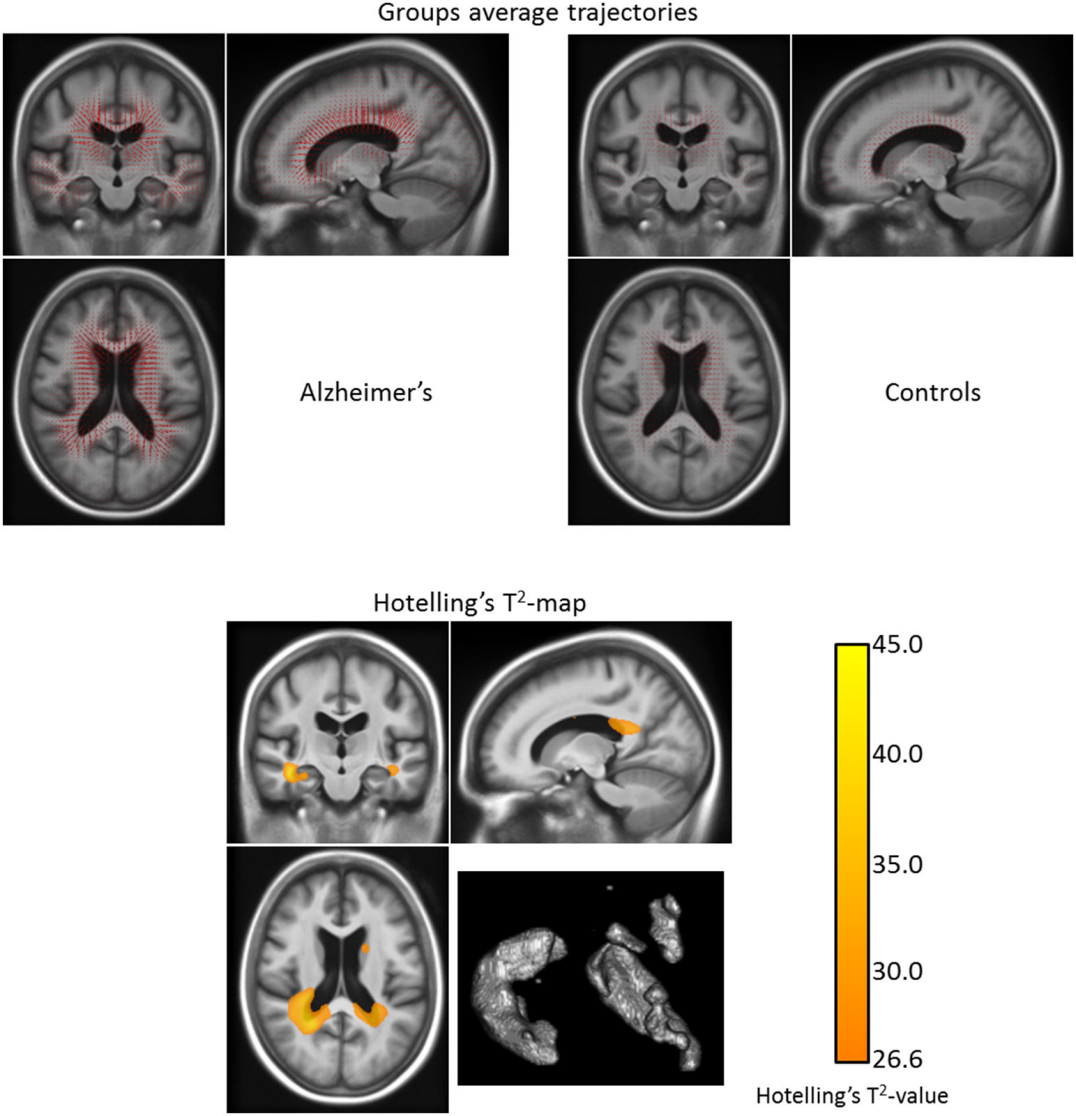
**Top:** Group longitudinal trajectories for the patients with Alzheimer's disease and the healthy control group (obtained with the LLDF method). We can see that the mean trajectory for the demented group has a higher magnitude than the control one. **Bottom:** Corrected *T*^2^-map for the longitudinal trajectories differences between the patients with Alzheimer's disease and the healthy control group (for the LLDF method) on one slice. The statistical differences between the demented and the control groups are located in the lateral ventricles, in the temporal horn of the ventricles, in the hippocampi, and in the caudate nuclei. The volume of the regions of statistical significant differences is 41.0 cm^3^. (The Hoteling's *T*^2^-test was corrected for multiple testing using 5000 permutations and the map is thresholded for a corrected *p*-value of 0.05).

Finally, we illustrate the main advantage of the LLDF: by using the parallel transport in our pipeline it is then possible to perform statistics directly on the subject-specific longitudinal trajectories. We therefore perform a multivariate Hoteling's two-sample *T*^2^-test to show the group-wise differences between the modeled subject-specific longitudinal trajectories of the Alzheimer's patients group and the healthy control group—obtained using the confidence mask. The null hypothesis is that there exists no difference between the mean of the two groups. We corrected for multiple testing using 5000 permutations and we limited the test to the brain mask. The resulting *T*^2^-map thresholded for a corrected *p*-value of 0.05 can be seen on Figure 10,^7^.

We can see that the statistical differences between the demented and the control groups are located in the lateral ventricles, in the temporal horn of the ventricles, in the hippocampi and, in the caudate nuclei. The volume of the regions of statistical significant differences is larger than the one found using the univariate test: 41.0 cm^3^. The observed differences in the statistically significant regions between the univariate *t*-test (cf. Section 3.2.1) and the multivariate Hoteling's *T*^2^-test can be explained by the fact that in the first case the study is restricted to the volumetry only whereas in the second case it focuses on the displacement field—which in addition to the volumetry also includes translations and rotations. With this difference in mind, we can say that the patterns found in the two tests are coherent. For example concerning the caudate nuclei, although there is no statistically significant difference in the volume changes between the patients with Alzheimer's and the healthy subjects, there exist statistically significant differences in the displacements of the caudate nuclei between the two groups.

## 4. Conclusion and discussion

We proposed and detailed a new processing pipeline^8^ for the longitudinal analysis of image data series. It is based on freely available software and tools so that anyone can reproduce our study, use this pipeline to replicate and verify findings conducted with other pipelines or use it to perform new studies. Moreover, we also implemented a masking of the similarity term in the non-linear registration (with a formulation that ensures symmetry). It enhances the robustness of the registration results with respect to intensity artifacts in the boundary of the brain, thereby increasing the sensitivity of the statistical studies done on the longitudinal deformations. We finally showed on an open-access database that the results obtained with this pipeline are consistent with the findings from the literature.

The use of the parallel transport in our pipeline enables us to perform both standard univariate analysis on a scalar map and also statistics directly on the SVFs as illustrated by the multivariate Hoteling's *T*^2^-test. Therefore, changes other than the ones linked to volumetry (like rotations or translations of the brain structures) could be studied. Concerning the confidence mask, initializing it with probabilistic masks of the fixed and moving image (instead of binary ones) could be used to take into account the uncertainty linked to the skull-stripping at the brain boundaries. However, in our experiment the use of binary masks was sufficient to increase the sensitivity of the statistical group-wise analysis while not decreasing the specificity. Intensities artifacts inside the brain such as prominent blood vessels could also be incorporated in the confidence mask if a blood vessels segmentation was available.

The most important issue for the longitudinal processing pipeline is related to the asymmetry biases (Ridgway et al., 2015) that need to be avoided in the processing. Two types of asymmetries can be distinguished. The first one, described in Reuter and Fischl (2011) and Yushkevich et al. (2010), is introduced by the resampling of all the follow-up images except the baseline. In our case, all the images (including the baseline image *I*_0_ at *t* = 0) are resampled once and only once in the common reference space. In the case of the follow-up images, the transformation used to resample the image is the combination of a rigid and an affine transformation (cf. Section 2.2.3), whereas in the case of the baseline image, we use the subject to reference space affine transformation only. This aspect of the pipeline has some similarity to that of Rohrer et al. (2013)—where again, some repeated interpolations are avoided, while other interpolations are symmetric by virtue of being in MNI space rather than in the native baseline space. It could be possible to go one step further and to avoid any explicit interpolation by initializing the non-linear registration (in the LCC log-Demons software) with the combined affine/rigid transformation using the software parameter:—initial-linear-transform. However, this would still imply an implicit internal resampling and in this case we would no longer follow the assumption made in LDDMM and the SVF framework that all the field tends toward zero when we get away from the center of the image (i.e., beyond the borders of the image). In practice, we observe edge-effects and a proper way to deal with the problem should be to revise the LCC log-Demons algorithm in order to explicitly handle the two transformations separately and make sure that the criterion (and the discretization) would be affine invariant.

The second type of bias is related to the non-centrality of the time point where the subject longitudinal deformations are computed (also referenced as favoring a particular time point). Several non-stationary velocity fields-based methods (LDDMM) have taken great care of that (Avants et al., 2011; Niethammer et al., 2011; Ashburner and Ridgway, 2013). In these methods, the initial velocity (or equivalently the momentum map) is different at different time points along a geodesic. In that case, for more than two time points, it is necessary to choose a time point for the subject-specific template, and this time point is generally the average (or median) of the observed time points. The momentum maps (from the template to all the time points) can then be compared in the template reference space only. In the stationary velocity field framework, the velocity field is—by definition—stationary. Thus, the SVF resulting from the registration is the same all along the trajectory: it is not expressed in material coordinates at a specific time point but in Eulerian coordinates which are not attached to a given time point. Therefore, in the symmetric LCC log-Demons any subject time point can be chosen to perform the pairwise registrations without needing a subject-specific template. Moreover, the annualized log-Jacobian map is valid for all time points even if its value for a material point changes with time along its trajectory. Finally, even if each registration is fundamentally pairwise, the effect of the multiple time points is taken care of using the fully symmetric linear model in time described in Section 2.3.3. This model uses all the possible combinations of SVFs in order to avoid favoring any specific time point. Notice that this approach is sub-optimal with unbalanced data where large variations exist in the number of time points *N*_*i*_ between the subjects. This can be corrected using methods like the one described in Guillaume et al. (2014). However, in the study presented here, only 13 subjects out of 136 had more than three time points. The majority had two or three time points which did not unbalance the data too much.

Apart from the bias, one can wonder what would be the best method between LDDMM and the SVF framework. At first sight, LDDMM might appear as a better theoretical model for an elastic mechanical deformation since it is based on the conservation of the Hamiltonian. However, it is not completely clear that the longitudinal evolution of a brain (intra-subject) is an elastic deformation that conserves the energy. Moreover, in practice Lorenzi and Pennec (2013) showed that for the longitudinal registration the differences between the two methods are very subtle and the stationary velocity field framework can be used.

## Author contributions

MH has worked on the design, implementation, and experimental study of the pipeline. ML, NA, and XP have co-supervised him in the design and revision of the work, and they gave their final approval of the version to be published.

### Conflict of interest statement

The authors declare that the research was conducted in the absence of any commercial or financial relationships that could be construed as a potential conflict of interest.

## Footnotes

^1^http://sourceforge.net/projects/neuropipedream/

^2^https://github.com/Inria-Visages/Anima-Public/wiki

^3^http://surfer.nmr.mgh.harvard.edu

^4^http://afni.nimh.nih.gov/

^5^The log-Jacobian maps (for OAS2_0002 and the different groups) are available on NeuroVault (Gorgolewski et al., 2015) at http://neurovault.org/collections/YBADDEIH/.

^6^The t-maps as well as the group difference and estimated variance maps for the three methods are available at http://neurovault.org/collections/YBADDEIH/.

^7^The *T*^2^-map is available at http://neurovault.org/collections/YBADDEIH/.

^8^The whole pipeline will be released as a complement of the already available LCC log-Demons software.
