# Supplementary material for: Longitudinal Analysis of Image Time Series with Diffeomorphic Deformations: A Computational Framework Based on Stationary Velocity Fields
Source: Front Neurosci. 2016 Jun 3;10:236. doi: 10.3389/fnins.2016.00236 (PMC4891339; doi:10.3389/fnins.2016.00236)
Supplement: Supplementary file 1 [file Presentation1.pdf]

Appendix — Longitudinal Analysis of Image Time Series with Diffeomorphic Deformations: a Computational Framework based on Stationary Velocity Fields

Optimisation of the LCC log-Demons energy with a confidence mask

We detail here the optimisation of the LCC log-Demons energy function (defined Section 2.3.2) with a confidence mask ω and the closed-form solution of the update δv. We consider the first part of the optimisation where E_{Sim} is minimised with respect to v_c, the auxiliary SVF:

$$E_{Sim}(\delta v, I_i, I_j) = -\frac{\omega}{\sigma_i^2}\rho'^2(\delta v, I_i, I_j) + \frac{1}{\sigma_x^2}||\delta v||^2. \quad (3)$$

We refer here to the optimisation of the LCC-correspondence of Lorenzi et al. (2013), using Λ from equation (2) we know that the squared LCC $(\rho^{\delta v})^2$ can be approximated by

$$(\rho^{\delta v})^2 \approx (\rho + \frac{\rho}{2}\Lambda\delta v)^2 = \rho^2(1 + \frac{1}{2}\Lambda\delta v + \frac{1}{4}\delta v^T\Lambda^T\Lambda\delta v).$$

Its gradient is $D((\rho^{\delta v})^2) = \frac{\rho^2}{2}\Lambda$, and its Hessian is $H((\rho^{\delta v})^2) = \frac{\rho^2}{4}\Lambda^T\Lambda$.

Therefore the optimal of the energy (equation (3)) is given by :

$$\left(\omega H((\rho^{\delta v})^2) + \frac{\sigma_i^2}{\sigma_x^2}Id\right)\delta v = -\omega D((\rho^{\delta v})^2),$$

and we can deduce that the solution is :

$$\delta v = \begin{cases} -\frac{2\Lambda}{||\Lambda||^2 + \frac{1}{\omega}\frac{4}{\rho^2}\frac{\sigma_i^2}{\sigma_x^2}}, \text{ if } \omega > 0 \\ 0, \text{ if } \omega = 0 \end{cases}$$

List and information about the OASIS Subjects Used

For the reader to be able to replicate the presented results, the list of OASIS subjects we used can be downloaded here: `http://www-sop.inria.fr/teams/asclepios/data/Pipeline/lists/OASIS_List_Subjects.csv`. We kept the complete set of demented and non-demented subjects and only excluded the subjects who converted - from non-demented to demented - during the study. The images can be found here (we used the mpr-1 acquisitions only): `http://www.oasis-brains.org/app/template/Tools.vm;jsessionid=9A56E751939B1DAE41049A46BBAAFB3E#services`. Socio-demographic and clinical information on the sample can be found Table 1.

Group	Non-Demented	Demented
Age at baseline (years)	75 ± 8	75 ± 7
Female/Male	50/22	28/36
Education (years)	15 ± 3	14 ± 3
Mini Mental State Examination (MMSE)	29 ± 1	25 ± 3

Table 1: Socio-demographic and clinical information of the study cohort

We notice that there is a statistically significant (by Fisher exact test) gender imbalance across the two groups (50/72 vs. 28/64). These gender effects could bias the study and were not accounted for. However, since we benchmark (cf. Section 3.2.2) the three methods - LLDF, Pipeline Head, and Pipeline Skull-strip - using the same sample, this gender imbalance should not be an issue.

Versions of the Software Used and Links to Download Them

In the following list, the reader can find the version of the software used and the links to download them so that he can replicate the processing pipeline (all the mentioned software were installed on Linux):

- FSL version 5.0.2.1: `http://fsl.fmrib.ox.ac.uk/fsldownloads/`
- ANTS version 1.9.v4: `https://github.com/stnava/ANTs/tarball/master`
- Robex version 1.0: `https://www.nitrc.org/projects/robex`
- LCC log-Demons with confidence mask: `http://www-sop.inria.fr/teams/asclepios/software/LCClogDemons/LCClogDemonsV1.2.tar.gz`
- Parallel transport (Pole ladder): `http://www-sop.inria.fr/teams/asclepios/software/LCClogDemons/Ladder.tar.gz`
- Stationary Velocity Field Tools: `http://www-sop.inria.fr/teams/asclepios/software/LCClogDemons/SVF_Tools.tar.gz`
- SPM8: `http://www.fil.ion.ucl.ac.uk/spm/software/spm8/`
